# An explainable vision transformer model with transfer learning for accurate bean leaf disease classification

**DOI:** 10.1038/s41598-026-41723-9

**Published:** 2026-02-24

**Authors:** Saiprasad Potharaju, Arun Singh, Dalwinder Singh, Swapnali N. Tambe, Prasad MVV Kantipudi, B. Kiranmai

**Affiliations:** 1 Department of CSE, Symbiosis Institute of Technology, Symbiosis International (Deemed University), Pune, India; 2 School of Computer Science and Engineering, Lovely Professional University, Phagwara, Punjab India; 3 Department of Information Technology, K. K.Wagh Institute of Engineering Education & Research, Nashik, MH India; 4 Department of ETC, Symbiosis Institute of Technology, Symbiosis International (Deemed University), Pune, India; 5 Dept of CSE(DS), Sreyas Institute of Engineering and Technology Hyderabad, Hyderabad, India

**Keywords:** Bean Leaf Disease, Vision Transformer, Transfer Learning, Explainable AI, GradCAM++, Deep Learning, Computational biology and bioinformatics, Engineering, Mathematics and computing

## Abstract

Early identification of bean leaf diseases, particularly Angular Leaf Spot and Bean Rust, is vital for ensuring crop productivity and global food security, especially within smallholder farming systems where disease outbreaks can rapidly escalate and cause severe yield losses. Conventional disease identification through visual inspection is labor-intensive, subjective, and highly dependent on expert knowledge, making it impractical for large-scale agricultural monitoring. Although recent deep learning-based approaches have demonstrated impressive accuracy in plant disease classification, their inherent “black-box” nature significantly limits real-world adoption, as farmers and agronomists often lack the ability to understand, trust, or act upon unexplained predictions. To address these challenges, this study proposes an automated and explainable disease diagnostic framework based on a Vision Transformer (ViT-B/16) architecture optimized through transfer learning from ImageNet. Unlike traditional convolutional neural networks that primarily focus on localized features, the Vision Transformer processes images as a sequence of flattened patches and leverages self-attention mechanisms to capture long-range dependencies and global contextual patterns across the entire leaf surface. This global representation enables the model to detect subtle and spatially distributed disease symptoms that are often overlooked by CNN-based approaches. To further enhance transparency and interpretability, GradCAM + + is integrated into the framework as an explainable artificial intelligence (XAI) mechanism. This method generates class-specific heatmaps that visually highlight the exact pathological regions influencing the model’s predictions, thereby establishing a human-interpretable validation loop for farmers, agronomists, and domain experts. The proposed framework was evaluated on the publicly available I-Bean dataset, achieving a validation accuracy of 97.52% along with strong precision, recall, and F1-score performance. The generated GradCAM + + visualizations consistently demonstrate the model’s sensitivity to true diseased regions, reinforcing both the reliability and trustworthiness of its predictions. By combining high-capacity global feature learning with visual explainability, the proposed approach offers a scalable, transparent, and practical solution for real-world precision agriculture. This framework not only enhances diagnostic accuracy but also bridges the critical gap between model performance and user trust, enabling informed decision-making and timely disease management in modern farming environments.

## Introduction

**Fig. 1:**
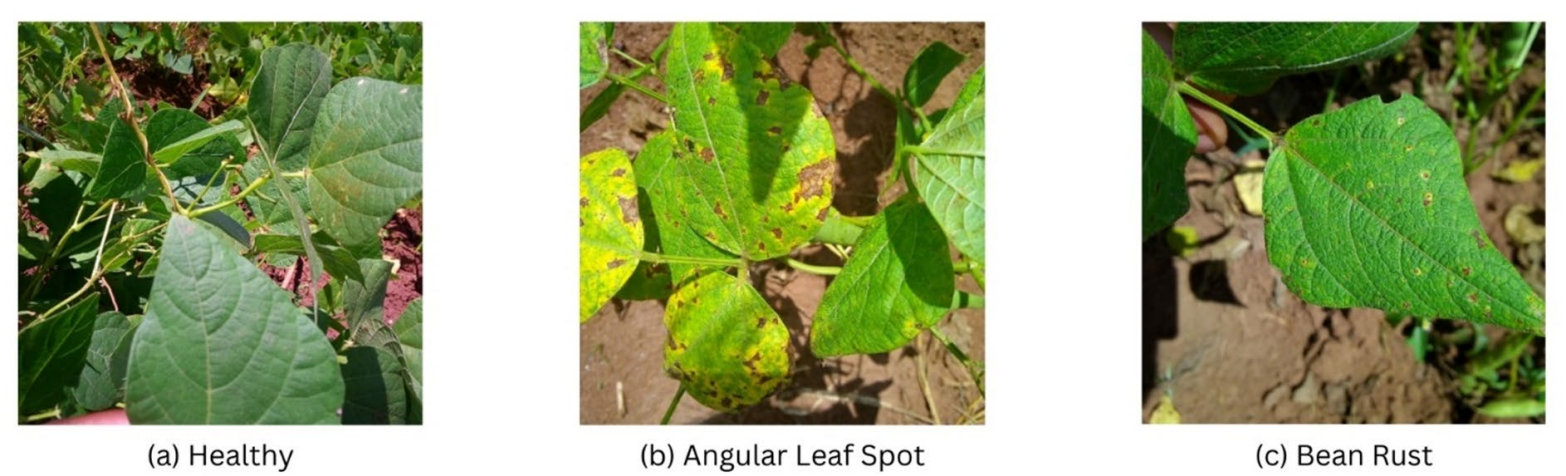
Image samples of (**a**) Healthy, (**b**) Angular Leaf Spot, and (**c**) Bean Rust leaves.

Phaseolus vulgaris L. (common bean) is one of the foundations of food security world over and forms an important part of sustainable food systems. Being a main source of dietary protein and fiber in millions of people, especially in developing countries, its stable cultivation is needed to achieve both nutritional and financial security. Nevertheless, the potential of one of the most important crops is always undermined by a series of pathogenic diseases capable of ruining harvests. Fungal pathogens that cause diseases like the Angular Leaf Spot (ALS) and Bean Rust are among the most virulent shown above in Fig. 1. The diseases impair photosynthetic ability of the plant causing defoliation, low quality of pods and losses by the farmers are high. This issue is further aggravated by the growing interchange and fast development of these pathogens, and hence requires dynamic and sophisticated methods of management. This has made it a keen priority to research on strong means of detection including challenging hybrid deep leaning models to be used in contemporary farming^1^.

Traditionally, the first line of crop disease prophylaxis has been a manual control of crops by agronomists and plant pathologists who have to visit and scan the field on a regular basis. Although this classic approach is useful, it is marred with underlying shortcomings. It is extremely time consuming and hence cannot be welcomed in the mass production of agriculture and its diagnostic specificity and sensitivity can change drastically according to the individual expertise and thus results in confused and chance-against-chancereadings. Logistical delays that are occasioned by manual surveys usually cause the policy window of intervention to be missed. In order to conquer these challenges, computer vision and artificial intelligence tools in the agricultural technology business have been turning more and more to the field. Such technologies provide objective, scalable, and unlimited crop health monitoring technique. The idea of explainable deep learning networks, including the ability to combine different architectures such as Transformers and CNNs, is a tremendous step; the resulting system is not only supposed to predict a disease, but also to explain the specific factors that prompted the verdict in a rationale way^2^. Moreover, the potential of machine learning algorithms to correctly predict the severity of the disease based on basic photographs is only going to put farmers ahead on the way they cope with crops management and distribution of resources^3^.

The integration of Artificial Intelligence (AI) and Computer Vision (CV) has become a cornerstone for ensuring food security, particularly in developing nations where agriculture is a vital economic sector. Advanced techniques are now applied across the entire agricultural lifecycle—pre-harvest, harvest, and post-harvest—ranging from crop disease detection and yield estimation to real-time monitoring and post-harvest quality control. Such multi-phase automation is instrumental in minimizing labor while significantly improving crop quality and reducing losses^37^.

Machine learning-based image-based autonomous crop disease detection is a technique that has come out of the research niche into a major research area^4^. Earlier systems invariably relied on classical image processing methods and hand-designed features, and needed extensive knowledge of the domain, and tended to be brittle to changes of lighting or background. The paradigm was replaced in the emergence of deep learning and, in particular, Convolutional Neural Networks (CNNs). CNNs are able to learn how to represent features hierarchically, in a direct fashion on the basis of pixel data, thus becoming the workhorse in terms of image classification tasks. This has led to the invention of new models, such as generalized methods of cross-crop disease detection^5^, and stacked CNN ensembles based on transfer learning to further increase performance^6^. Recently the research has shifted towards a study on how to solve the complexities of the in-field detection via powerful object detection frameworks such as YOLO that accommodate busy backgrounds and changing environmental levels^7,8^. The best performing models still have a perennial headache, the so called black box problem. In a bid to ensure adoption and trust by the end-users, there has been an imminent need to have systems that are not only precise, but also transparent. In its turn the methodologies that allow to obtain explainable answers about the way the model makes decision are getting more and more important^9^.

Significant strides have been made in identifying fungal diseases across diverse regional datasets and varying climates. For instance, modified ResNeXt variants of convolutional neural networks (CNNs) have demonstrated high accuracy (98.92%) in predicting fungal infections such as spots, scabs, and rusts in fruit crops like apple, guava, and custard apple. Furthermore, end-to-end pipelines utilizing pretrained ResNet-50 backbones and specialized segmentation models like SegLearner have enabled precise assessment of disease severity through pixel counting, which is essential for limiting the spread of infestations^38^. Beyond disease detection, innovative automated systems such as 3SW-Net, which combine SLIC segmentation and HOG feature extraction, have achieved near-perfect recall in weed detection, further promoting sustainable resource management^39^.

While significant achievements have been documented in high-accuracy classification and segmentation for various crops, several challenges remain unaddressed. Current research highlights persistent hurdles in model generalization across varying field conditions, data scarcity for niche pathologies, and the lack of transparent reasoning in complex models^37^. Many existing algorithms focus on specific regions or tasks—such as severity assessment or weed detection—but often fail to provide the end-to-end explainability required for farmer trust in high-stakes agricultural decisions. This research specifically targets these remaining gaps by merging the global feature extraction power of Vision Transformers with explicit XAI techniques to address the “black-box” limitations of prior approaches^38^.

The research problem has a root based on the twofold challenge of professional and precise diagnosis of the bean leaf diseases, and the second challenge is that the diagnostic tool must be reliable to the end-users. Conventional manual examination by specialists is immoderately colossal, subjective and labor burdensome to adequately manage huge-scale crop. Although the modern deep learning models may show a great accuracy, they frequently represent the so-called black boxes, which deliver a prediction without reasoning. The absence of transparency is a setback to farmers since they need a clear and comprehensible ground of making decisive decisions. Thus, the existing issue is that no tool exists that is fast, highly accurate and explainable, and a desperate need to find a reliable and trustworthy fully automated tool in practical agriculture occurs.

The aim of the proposed study is to design and build a highly accurate deep learning model to classify common bean leaf diseases that can be critiqued and use both accuracy, and understandability as priorities. The given work is going to solve this crucial constraint of the agricultural AI as black box predictor by suggesting a new solution as a combination of a strong Vision Transformer and an explainable AI approach, thus offering a clear, trustworthy resolution. The method is based on feature extraction ability using transfer learning technique and the results on a publicly available public dataset of leaf images collected in the field and annotated by agricultural experts is considered.

The innovation about the paper is the automated system suggested by establishing a synergistic combination of a Vision Transformer (ViT) model with a GradCAM + + explainable AI (XAI) method to classify the bean leaf disease. The ViT architecture provides excellent predictive accuracy due to using the transfer learning paradigm by relying on the pretrained ImageNet weights. The imperative inclusion of GradCAM + + transforms the model decisions clear by producing visual heat.*; the model therefore provided a unified program, which is accurate and reliable.

Despite advancements in automated detection, most systems fail to reconcile high-dimensional feature extraction with human-readable evidence. Existing hybrid models often prioritize local textures (CNN-based) or global structures (Transformer-based) in isolation. Our work addresses this by implementing a unified pipeline that specifically leverages the ‘long-range dependency’ of ViT to detect early-stage, diffuse lesions while utilizing GradCAM + + to validate that the model is attending to actual biological symptoms rather than background noise.

The rest of the paper is structured as follows: section II offers a survey of literature of the related works in the area of plant disease classification. Section III gives a detailed account of the data set, methods of preprocessing and analytical tools of our analysis. Section IV explains the intended methodology, about the Vision Transformer architecture and the incorporation of the GradCAM++. Section V shows experiment results, performance evaluation and discussion of the results. The conclusion or Third Section VI eventually wraps up the paper and proposes future studies.

## Related works

Academic setting of the field of automated plant disease recognition is very rich and evolving with scientists constantly expanding the capabilities of deep learning only to reach new levels of accuracy and usability. The step further in terms of not ordinary models is manifested in the research of the advanced learning paradigms. An example of this can be an integration of deep reinforcement learning with transfer learning in hybrid frameworks, which has been successfully used in the case of tomato plant diseases, demonstrating that new compounds of AI techniques can produce more versatile and sturdy models^10^. This area of operation is also overtaking traditional usage on RGB imagery. The use of spectral data processing and deep learning indicates that integrating different data types may allow capturing more discriminating features resulting in a higher accuracy of the estimated condition such as Adzuki bean rust^11^. Moreover, AI use is no longer just binary classification but can now be used in more greyscale duties like the level of severity of Cercospora leaf spot in mung beans, which can give the farmer even more to act on and predict production^12^.

The essence of this technological shift is the fact that it replaces classical machine learning is based on hand-engineered features with the paradigm of automatic feature extraction championed by deep learning. Such transition has offered a much powerful and adaptable plant protection and biosecurity arsenal^13^. Real-world deployment is a major trend that was evident in recent literature and stimulated studies on edge AI systems. By creating an algorithm based on the detection of products, such as beans of the coffee plant, which will work in conditions of a low-power edge device, researchers are trying to bring real-time analytical functions to the hands of farmers directly, without being tied to a stable internet connection and cloud resources^14^. The basic effectiveness of DL in plant diseases classification has proved itself, and was confirmed in a countless number of research studies and classes of crops, making it one of the core digital agriculture technologies^15^. As brittleness of models is a ubiquitous issue (as a model has been trained on one set and it fails on another one), multi-dataset training approaches are rising in popularity among researchers. These methods seek to construct more generalized and more robust models by exposing the models to a broader range of plants and environments and imaging equipment to construct systems that are robust across broader agricultural scenarios^16^.

Although the use of Convolutional Neural Networks (CNNs) has been prevalent in computer vision over the previous years, it can be a set-back or a constraint since it has the inductive bias of being locally directed. CNNs are good at identifying textures and patterns with a limited receptive field but could fail at modeling long-range dependencies on all pixels in an image. This has contributed to the development of use of Transformer architectures that were originally developed to process sequences in natural language. Transformers and transfer learning used to detect tomato leaf diseases in its early stages have already been implemented successfully, a phenomenon that demonstrates that the self-augmentation mechanism can adequately seize the global context and delicate and diffuse distributions of tomato disease symptoms on leaf surfaces, in many cases producing superior results when compared with their CNN analogues^17^.

The final challenge to any AI model in the agricultural field is how they work in the real and unstable field conditions. Efforts have been made analyzing this challenge by the use of powerful object detection models such as YOLO in determining the presence of diseases that are encountered in the common bean despite the presence of noisy backgrounds, varying lighting and the partial occlusion of the subjects^18^. Another vital obstacle is the ubiquitous problem of low data availability in niche areas of agriculture. The problem of limited data has been managed so that researchers have been able to artificially grow the numbers in small datasets using extensive data augmentation, effectively training deep learning models that could perform real-time detection without the necessity of massive data collection^19^. The push towards the applied on-site efforts is further illustrated by the research which deploys edge computing to introduce deep learning-based bean pathology detection at the field setting, which is a defining move to realizing the existence of timely and data-driven farm management capabilities^20^. Hand in hand with these innovations, deep convolutional neural network models are still in the process of being perfected in bean disease classification^21^, rigorous comparative analyses of various architectures are being conducted to benchmark performance to provide the most suitable measures to address a certain agricultural problem, which include different versions of YOLO^22^. The number of these pieces of work brings to the fore a distinct and sustained trend in the field; the direction of developing AI applications that can not only be highly accurate, but are also robust, deployable, and are eventually useful in actual agricultural practice.

The muscle underlying the framework is squarely addressing the major issues recognized by other resources through a synergistic union of a Vision Transformer (ViT) architecture and an explainable AI (XAI) component. First, it addresses a serious issue of the so-called black box that underlies most deep learning models by incorporating the GradCAM++. This also gives each individual classification open, pictorial explanations which builds user faith and goes beyond being predictive to be generative in giving keen insight. Second, using a Vision Transformer in our model, we manage to break the locality bias of CNNs. The self-attention mechanism of the ViTs is quite capable of identifying neither close nor extensive dependencies and global contextual patterns on the entire image of the leaf, which is essential in detecting subtle or far-flung disease manifestations. Lastly, transfer learning through a pretrained ViT on ImageNet means that the model already has a strong basis on feature extraction, hence it becomes possible to get an accuracy of more than 97.52% with a specialized dataset. This high-accuracy, architectural innovation, and explainability combination sets a new standard of viable and trustworthy solution ready to find use in agricultural practice, directly responding to the industry need of more powerful, reliable, and explainable diagnostic tools.

## Materials and methods

This section provides a highly detailed account of the experimental materials and methodological framework used in this research. A thorough description is provided to ensure the transparency and reproducibility of our study.

## Materials

### Dataset description

This research draws on such a strongly enhanced version of a freely distributed, publicly available dataset that was initially published by the AI Lab at Makerere University and was labeled by experts at Uganda National Crops Resources Research Institute (NaCRRI)^23,24^. To create an incredibly solid model that it will be in a one-shot learning position, the original training and validation sets were increased by about 40 times (with the augmentation mechanisms that are described in Section III-A-2). The outcome of this process was a much larger dataset that is almost balanced in the three classes. In order to provide a fair and direct comparison with previous papers that worked using the original dataset; the test subset of 128 images were maintained in the same original and un-augmented bank. The last, extended distribution of the experimenting dataset is provided in Table 1. Training high-capacity models such as Vision Transformers using augmented/enlarged datasets of this magnitude is one of the essential tactics to produce a state of the art performance and avoid overfitting^25^.

The images in the dataset are classified into three critical categories, each representing a distinct state of the bean plant’s health:

- Healthy: These are images of leaves that are free from any visible signs of disease, serving as the baseline or control class for the model.
- Angular Leaf Spot (ALS): Caused by the fungus *Phaeoisariopsis griseola*, this disease is characterized by necrotic lesions that are distinctly angular in shape, often bordered by the leaf’s veins.
- Bean Rust: Caused by the fungus *Uromyces appendiculatus*, this disease manifests as small, reddish-brown pustules that can cover the leaf surface, giving it a “rusty” appearance.

**Table 1:** Class-wise distribution of the original I-Bean dataset.

Set	Healthy(H)	Angular Leaf Spot (ALS)	Bean Rust (BR)	Total
Train	13,600	13,800	13,900	41,300
Validation	1760	1760	1800	5320

### Data preprocessing and augmentation

A multi-stage data preprocessing and augmentation pipeline was constructed using the *torchvision.transforms* library to prepare the images for the Vision Transformer model. This pipeline is critical for two reasons: standardizing the input data to match the model’s requirements and artificially expanding the training dataset to improve model generalization and prevent overfitting.

For the training set, a series of random geometric and color transformations was applied. This strategy introduces variability, teaching the model to become invariant to changes in orientation, position, and lighting. The specific augmentations were:

- RandomRotation(30): Simulates different camera angles by randomly rotating the image within a ± 30-degree range.
- RandomHorizontalFlip() & RandomVerticalFlip(): These teach the model orientational invariance, ensuring it can recognize a diseased leaf regardless of its orientation in the frame. Each is applied with a 50% probability.
- ColorJitter(): This randomly adjusts the image’s brightness, contrast, saturation, and hue, making the model more robust to the wide variations in lighting conditions encountered in the field (e.g., sunny vs. overcast days).

For all datasets (training, validation**)**, a uniform preprocessing sequence was applied to ensure consistency. This is not for augmentation but for standardization:

- Resize((224, 224)): The Vision Transformer (ViT-B/16) architecture has a fixed input resolution. This step resizes all images to 224 × 224 pixels to conform to this requirement.
- ToTensor(): This converts the images from the standard PIL format into PyTorch tensors, the fundamental data structure for computation within the framework.
- Normalize(): Each channel of the image tensor is normalized by subtracting the mean and dividing by the standard deviation of the ImageNet dataset (mean=[0.485, 0.456, 0.406], std=[0.229, 0.224, 0.225]). This centers the data distribution around zero and helps stabilize and accelerate the training process.

This comprehensive augmentation strategy is a cornerstone of modern deep learning practice, proven to significantly enhance the performance and robustness of deep neural networks, particularly when the original dataset size is limited^26^.

## Methodological framework

The experiments could be completed in a Python-based environment taking the assistance of the PyTorch deep learning framework. PyTorch was chosen because of the flexibility, a strong GPU acceleration environment, and a dynamic computation graph, which is very beneficial to research and development. The basic Vision Transformer was obtained using torchvision.models library, which contains the full catalog of the state-of-the-art models that can be easily found pretrained. This will help the transfer learning to be implemented, which is a major factor in our methodology. The torchcam library was used in our research in the important explainability dimension. This dedicated toolbox can provide effective and verified implementations of most varied Class Activation Mapping (CAM) algorithms. By abstracting the elaborate work of hooks and gradient manipulation involved, the package enables scholars to easily incorporate explainability techniques such as GradCAM + + in their pre-trained PyTorch networks. Modern computational science can not live without such high-level and well-sustained libraries since they speed up the experimental cycle and enable researchers to concentrate on the new scientific question instead of on the low-level implementation issues^27^.

**Algorithm 1:**
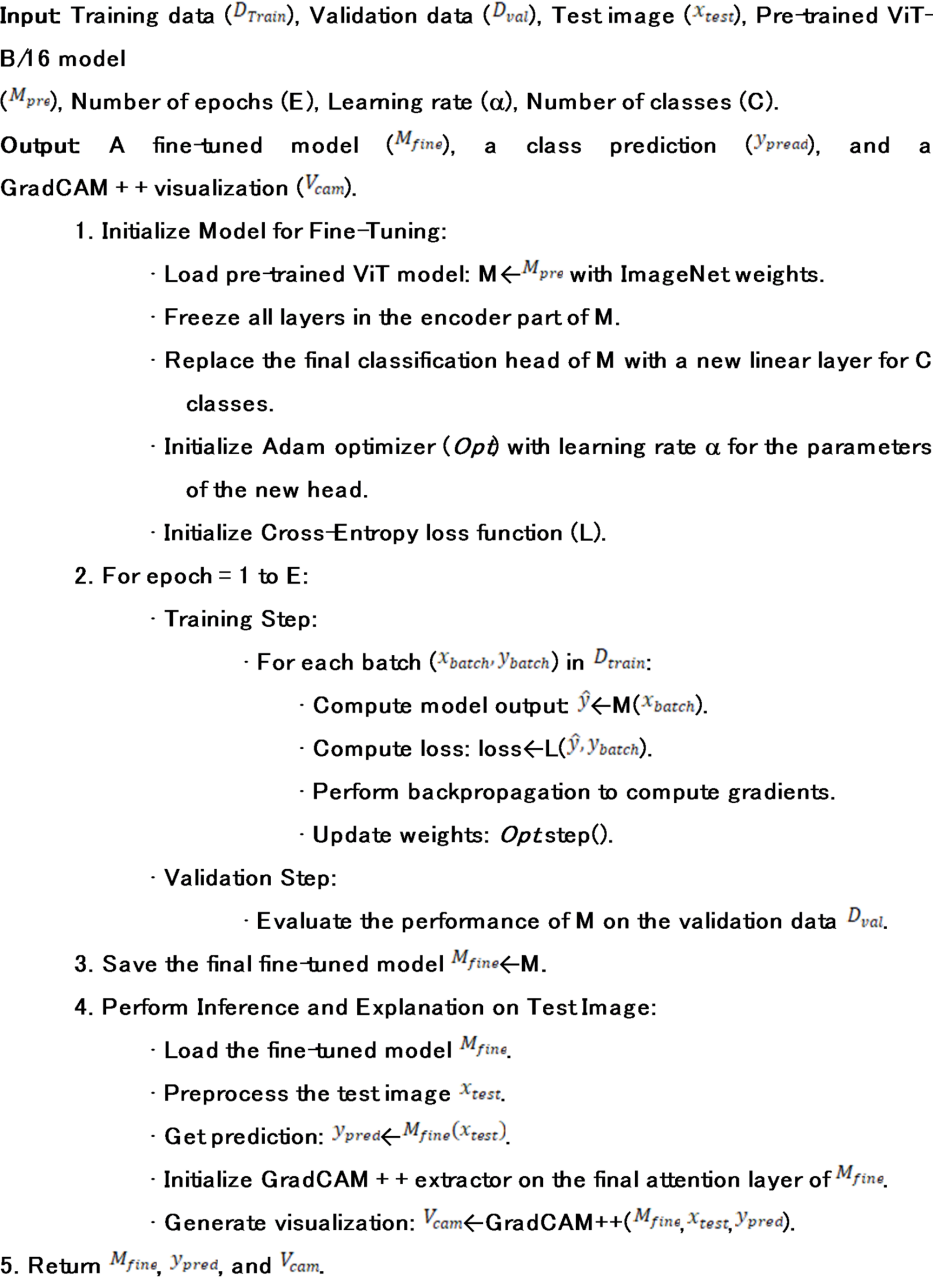
ViT Fine-Tuning and Explainability.

## proposed methodology

**Fig. 2:**
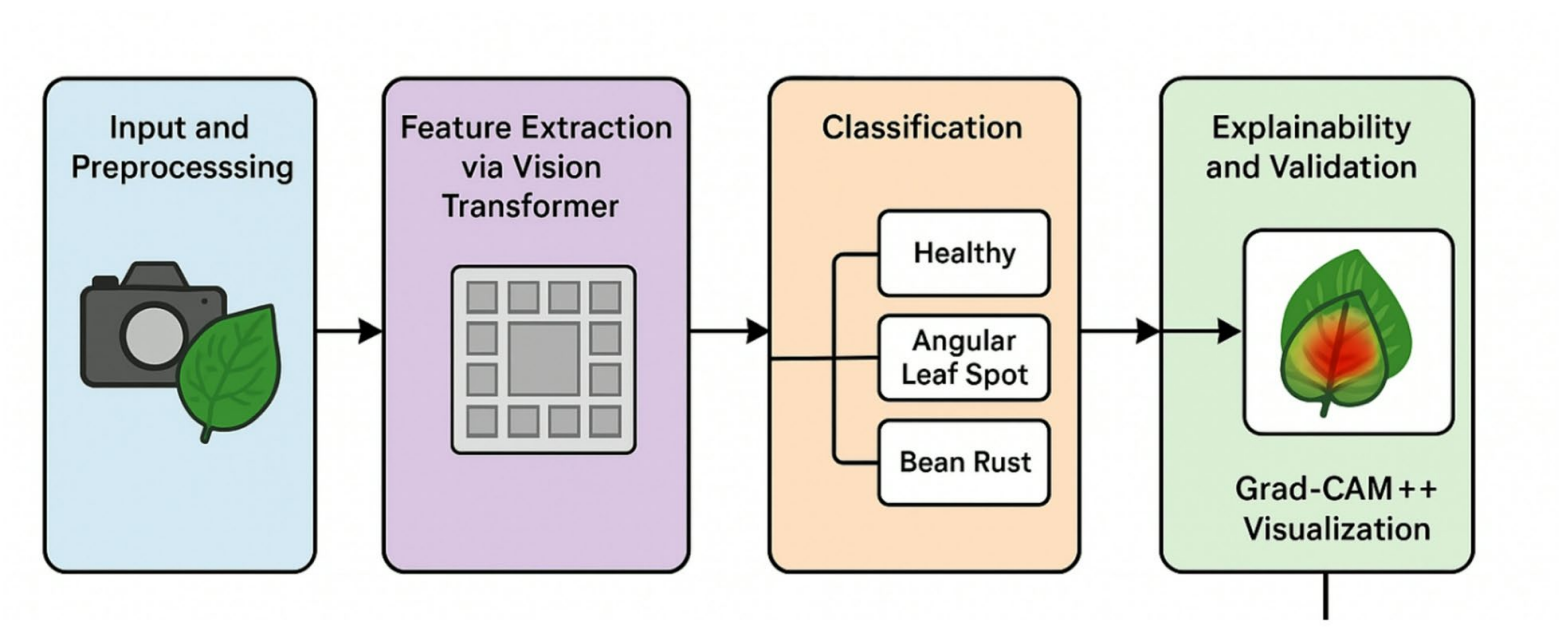
The overall framework pipeline for bean leaf disease classification.

**Fig. 3:**
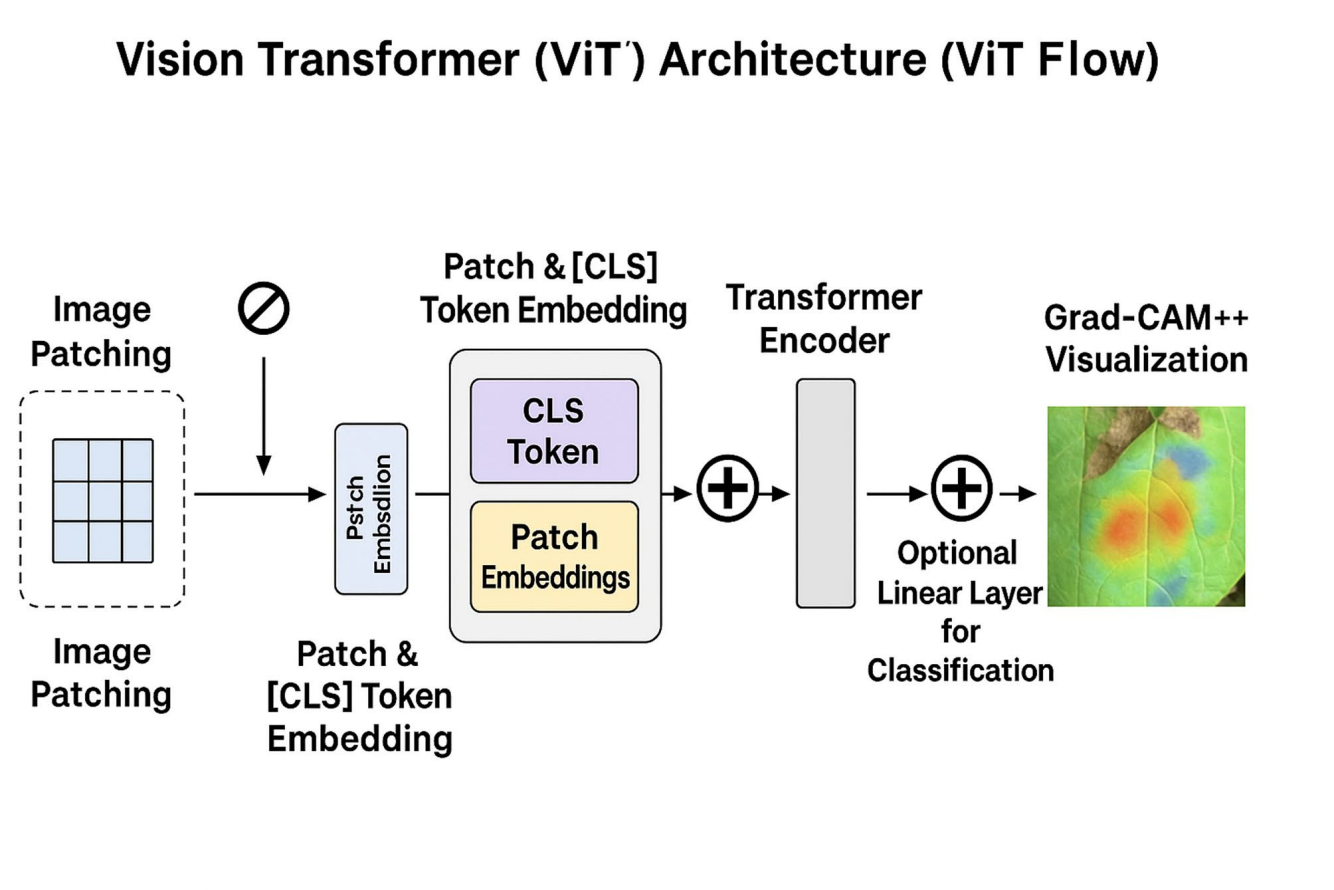
Vision Transformer (ViT) architecture pipeline for bean leaf disease classification.

This section provides a comprehensive technical exposition of the proposed framework, as shown in Fig. 2 for bean leaf disease classification. We detail the overall system architecture, the inner workings of the Vision Transformer model, the transfer learning strategy employed, and the integration of the explainability module as shown in Fig. 3.

### System model

The proposed system is defined as an end-to-end and human-in-the-loop, cross domain diagnostic pipeline, which goes beyond simple case-based reasoning and aspires to provide trustworthy, and evidence-based feedback to an end user, such as a farmer, or an agronomist. The workflow is structured in four stages, each of which builds on the previous one, to transform a raw image into a result that is actionable and interpretable.

1. Input and Preprocessing: This stage acts as a canonicalization gateway for all incoming data. An input image, that has been captured under a potentially arbitrary field conditions, is passed through the robust preprocessing and augmentation pipeline described in Section III-A. This stage is crucial for harmonizing the data so that the deep learning model can use it, as it is done at a fixed size, as well as pixel value distributions of the data. This stage also provides a form of controlled stochasticity through augmentation in order to improve model robustness for the training data.
2. Vision Transformer Feature Extraction: This image tensor, which has been standardized, is then entered into the heart of our model, a special Vision Transformer (ViT-B/16). In this stage, the encoder of the ViT, which has been trained on ImageNet, processes the image. He goes beyond the registers of elementary pixel data and retrieves advanced semantic data, which are abstract data that correspond to patterns and texture to be classified.
3. Classification: The last classification head is a one after which the Softmax function is applied. The learned representation which is the high-dimensional feature vector after being encapsulated is assigned a position in the feature space. The assigned position is subsequently translated into a probability for each of the three target classes: Healthy, Angular Leaf Spot, and Bean Rust.
4. Explainability and Validation. Simultaneously with classification, the framework activates the explainability module. The gradients that have been backpropagated from the prediction of the model get used for class activation with the GradCAM + + algorithm, which is one of the versions of GradCAM. The regions of the image that were used the most for the model to make the prediction are highlighted in the heatmap. The model is superimposed on the original image, allowing the user to see visually, and allowing the user to see a type of cycle of validation in which a user may “reasoning” of the model is correct and matches the evidence of the pathology.

This feature of providing visual evidence along with every prediction is a powerful builder of the confidence and understanding of the model behavior. The evidence also explains the preference for having a strong backbone architecture such as the ViT. While many top-performing models are based on a combination of powerful heuristic engineering and extensive pre-training, the architectural choice seems to be the single most important factor in determining ultimate performance in a head-to-head comparison of pre-trained models^28^.

### Vision transformer (ViT) architecture

The primary model we are working with is the Vision Transformer (ViT), which represents a shift from the convolutional model approaches that have dominated the computer vision field for years/decades. The ViT model, instead of localized convolutional filters, uses the transformer model from natural language processing (NLP) for image processing. It transforms the image into a sequence of patches (like one would do with a sentence into a sequence of words). With self-attention, the model can evaluate and attend to the information in the patches, and calculate the relations between any two patches—regardless of their spatial distance. This is particularly beneficial for the model to understand long distance or diffuse patterns of the disease. In our studies, we utilize the ViT-B/16 (“Base”) variant.

The ViT architecture processes the input x ∈ $$\:{R}^{H*W*C}$$ by reshaping it into N patches $$\:{x}_{p}\in\:{R}^{N*\left({P}^{2}.C\right)}$$. A learnable [class] token is prepended to the sequence, and 1D positional embeddings are added to maintain spatial integrity: $$\:{z}_{0}=\left[{x}_{class};{x}_{p}^{1}E,\dots\:;{x}_{p}^{N}E\right]+{E}_{pos}$$. The Transformer encoder then applies $$\:L$$ layers of Multi-Head Self-Attention (MHSA), allowing the model to weigh the importance of different leaf regions simultaneously.

1. Image patching and embedding.

The first step in the ViT pipeline is to deconstruct the 2D image into a 1D sequence. The input image $$\:x\in\:{R}^{H*W*C}$$ is partitioned into a grid of non-overlapping patches, each of size $$\:P*P$$. These patches are then flattened into vectors. For an input image of 224 × 224 and a patch size of 16 × 16 (as in ViT-B/16), this results in N=(224 × 224)/(16 × 16) = 196 patches. Each patch is a vector of size 16 × 16 × 3 = 768. These patch vectors are then linearly projected into a D-dimensional embedding space (for ViT-Base, D = 768) via a trainable weight matrix, E. To enable classification, a special learnable [class] token embedding $$\:\left({x}_{class}\right)$$ is prepended to this sequence. This token’s state at the output of the Transformer encoder will serve as the aggregate image representation for classification.

2. Positional embeddings.

The self-attention mechanism is inherently permutation-invariant; it does not have a built-in sense of order. If the patch embeddings were fed directly to the encoder, the model would perceive the image as an unordered “bag of patches.” To remedy this, learnable 1D positional embeddings $\:\left({E}_{pos}\right)$ are added to the patch embeddings. These positional embeddings encode the original spatial location of each patch, allowing the model to learn and utilize the image’s structure. The final input sequence to the encoder, $\:{z}_{0}$​, is formed as:

1 $$\:{z}_{0}=\left[{x}_{class};{x}_{p}^{1}E;{x}_{p}^{2}E;\dots\:;{x}_{p}^{N}E\right]+{E}_{pos}$$

2 $$\:{z}_{0}\in\:{R}^{\left(N+1\right)\mathrm{*}D},\:E\in\:{R}^{\left({P}^{2}.C\right)\mathrm{*}D},\:and\:{E}_{pos}\in\:{R}^{\left(N+1\right)\mathrm{*}D}$$.

Where.

3. Transformer encoder.

The essential part of the ViT is Transformer encoder which consists of a stack of L identical layers (L = 12 in ViT-Base). The general target of each layer is to improve by summing the information of other patches, found in the sequence, the description of each patch. The two most important sub-layers in a layer l include Multi-Head Self-Attention (MHSA) block and standard position-wise feed-forward network (MLP). In order to conveniently train such a deep network it has found that it is beneficial to apply Layer Normalization (LN) before each block, and use a residual connection after each block. The computation within each layer is thus:

3 $$\:{z}_{l}^{{\prime\:}}=MSA\left(LN\left({z}_{l-1}\right)\right)+{z}_{l-1}$$

4 $$\:{z}_{l}=MLP\left(LN\left({z}_{l}^{{\prime\:}}\right)\right)+{z}_{l}^{{\prime\:}}$$

The Transformer has an engine which is the MHSA block. It enables the model to learn difficult relations through actualization of attentional scores between every pair of patches. In theory, it produces a Query (Q) vector, Key (K) vector and Value (V) vector per patch. The attention score is calculated by dot producting a patch Query and all other patch Key. This ranking acts as the criteria as to the amount of attention a patch is supposed to accord to each other patch. The use of these scores is then to form a weighted conversion of all patch Values. This process can be run concurrently on different learned projections via the multiple “heads” which means that this model can capture the many possible types of relationships (e.g. textural, structural) simultaneously. The fundamental operation is the scaled dot-product attention:

5 $$\:Attention\left(Q,\:K,\:V\right)=softmax\left(\frac{\left(Q{K}^{T}\right)}{\surd\:\left({d}_{k}\right)}\right)V$$

where $\:{d}_{k}$ is the scaling factor, equal to the dimension of the key vectors. The MLP block, consisting of two linear layers with a GELU activation, serves to further process and transform the feature representations produced by the attention mechanism. The power of this architecture to learn complex data relationships has been highlighted in recent bibliometric analyses of deep learning trends in leaf disease detection^29^.

**Algorithm 2:**
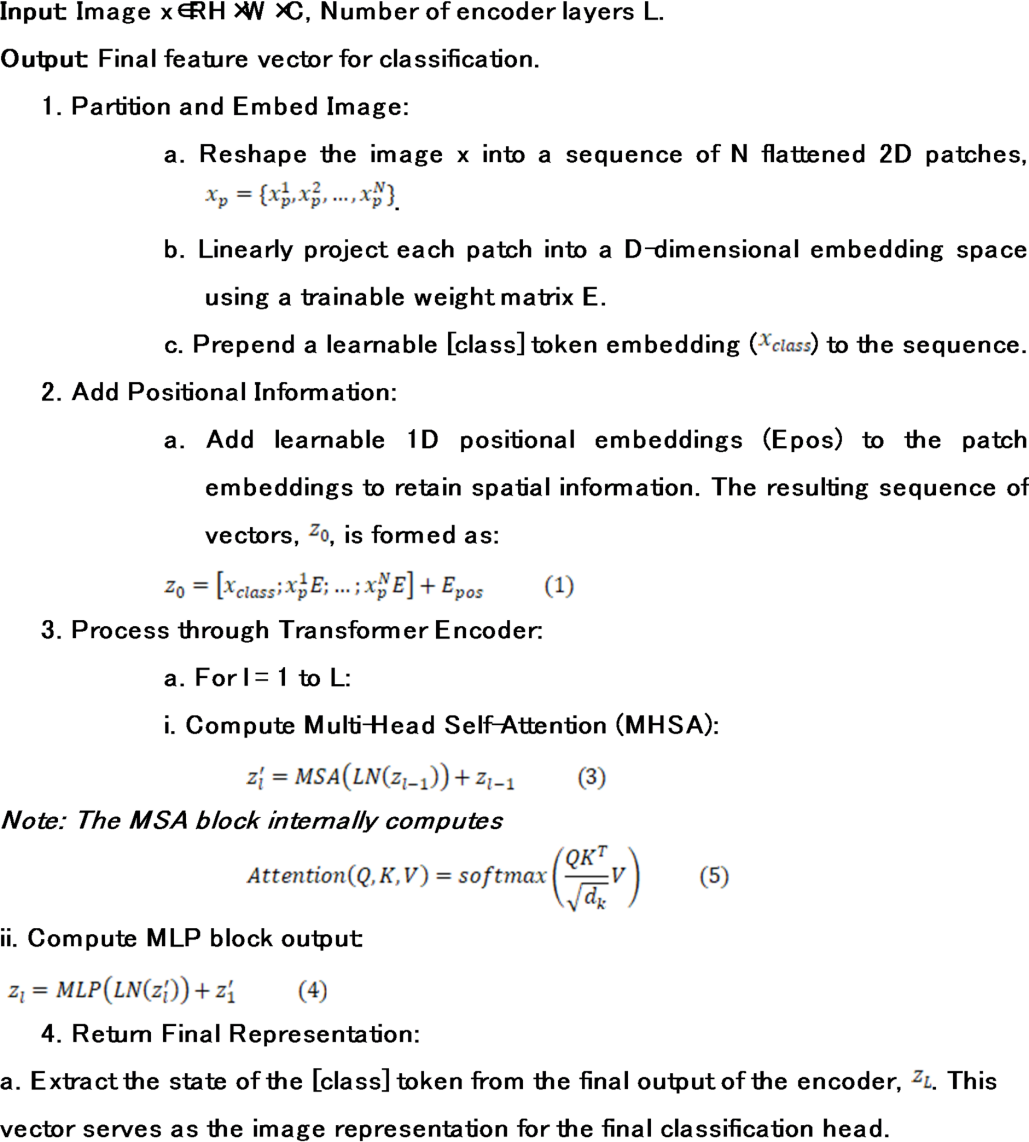
Vision Transformer (ViT) Forward Pass.

### Transfer learning for fine-tuning

Training such a data-hungry model, such as a Vision Transformer, using a random-initialized initialization involves such large datasets and may involve hundreds of millions of images. This is impractical on specialized areas such as the field of agriculture where the data scale is orders lower. This problem is circumvented through a transfer learning approach, which is one of the essential pillars of current applied deep learning. The methodology rests on the concept of feature reusability: by training a single model on a large heterogeneous dataset, such as ImageNet, researchers find that a wide range of features is subsequently learnt by the model, such as simple edges and colors, and more complex textures and shapes, which have general applicability to a wide variety of vision problems.

Our particular methodology for fine-tuning is planned and aimed at accomplishing the greatest possible knowledge transfer while reducing the potential for overfitting:

5. (1) Load Pretrained Model: The first step is loading the pretrained Vision Transformer (ViT) model at the layer resolution of B/16. Specifically for the ImageNet dataset, the model is pretrained on the version which contains one thousand images. This is the baseline foundation of the model and should be enough to equip the model with basic fundamental visual comprehension.
6. (2) Freeze Encoder Layers: We want to protect the learned information from being lost. Therefore, we will freeze the parameters of the twelve transformer layers of the encoder. This is done by setting requires_grad to the frozen layers as False so that during the training, these parameters will not be updated. This protects the model from catastrophic forgetting, which is essentially the model overwriting its general feature extractors because of an over-specialization to the narrow task at hand.
7. (3) Modify Classification Head: The model comes pre-configured with a classification head that predicts outputs for one thousand classes pertaining to the ImageNet dataset. This head is removed and in its place, a head that is designed for our specific task is put. This is an instance of a linear layer from the torch.nn class, and it is randomly initialized. For this particular instance, the head needs to reduce the dimension of the ViT’s of 768 [class] token to 3, with respect to the target classes (i.e. Healthy, ALS, and Bean Rust).
8. Fine-Tuning: The training process then focuses exclusively on optimizing the weights of this new, lightweight classification head. The frozen encoder acts as a fixed, powerful feature extractor. The model’s task is reduced from learning vision from scratch to simply learning how to map the rich features provided by the encoder to our specific set of class labels. This approach is computationally efficient and highly effective for domain adaptation^30^.

### Explainability with gradient-weighted class activation mapping (GradCAM++)

One of the main points that this study exhibits is that to be used in such a high-stakes application as agriculture, AI needs to be transparent. An accurate prediction means nothing when the person using a prediction cannot dissect or believe the reasoning behind the prediction. To deal with it, we seamlessly incorporate a prominent explainable AI (XAI) embedding Gradient-weighted Class Activation Mapping (GradCAM++), directly into our framework.

GradCAM + + and its earlier versions are meant to make it understand: what segments of the input photograph had the highest impact on the choice of a model to identify a specific category? It attains this by looking at the gradients of the final class score flowing back into the last high-level feature maps of the network (in our case, the attention maps of the last block of the Transformer). Areas having high gradients are recognized to be very crucial in the prediction. GradCAM + + advances the original Grad-CAM by adding a weighted combination of positive and negative gradients, resulting in more specific localization maps able to visualize whole objects and perform better with the multiple instances of the same category in a picture.

**Fig. 4:**
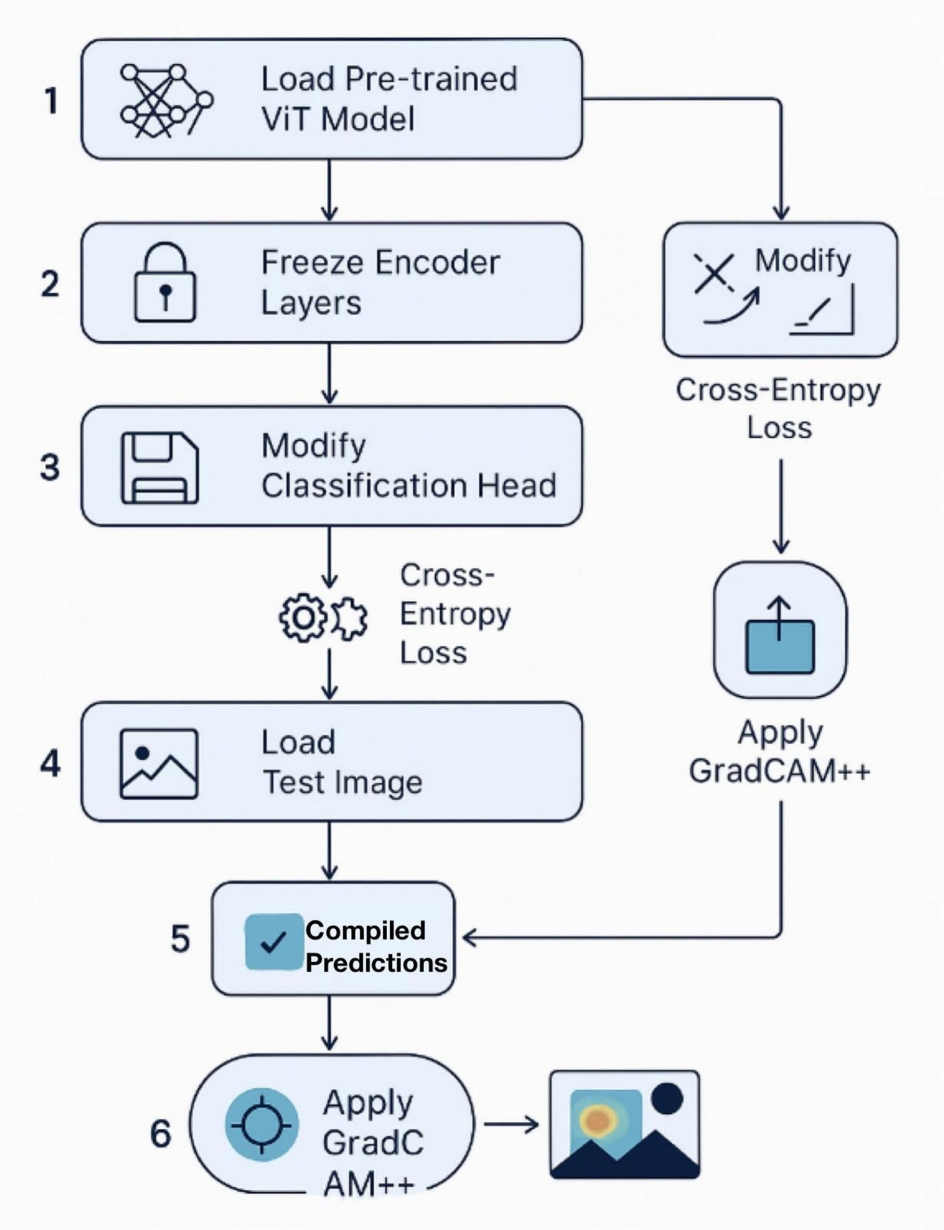
Flowchart illustrating the Explainable Vision Transformer (ViT) framework for bean leaf disease classification.

The operational flow for generating an explanation, as per Fig. 4, is as follows:

1. A forward pass is performed to obtain the model’s prediction for an input image.
2. The gradient of the predicted class score is computed with respect to the 3D feature maps of the target layer (the final attention block).
3. These gradients are used to calculate pixel-wise weights, which are then combined with the forward activation maps to produce a single, coarse heatmap.
4. This heatmap is upsampled to the original image’s resolution and color-coded (e.g., using a ‘jet’ colormap) to indicate importance, with hot colors like red signifying high influence and cool colors like blue signifying low influence.

**Algorithm 3:**
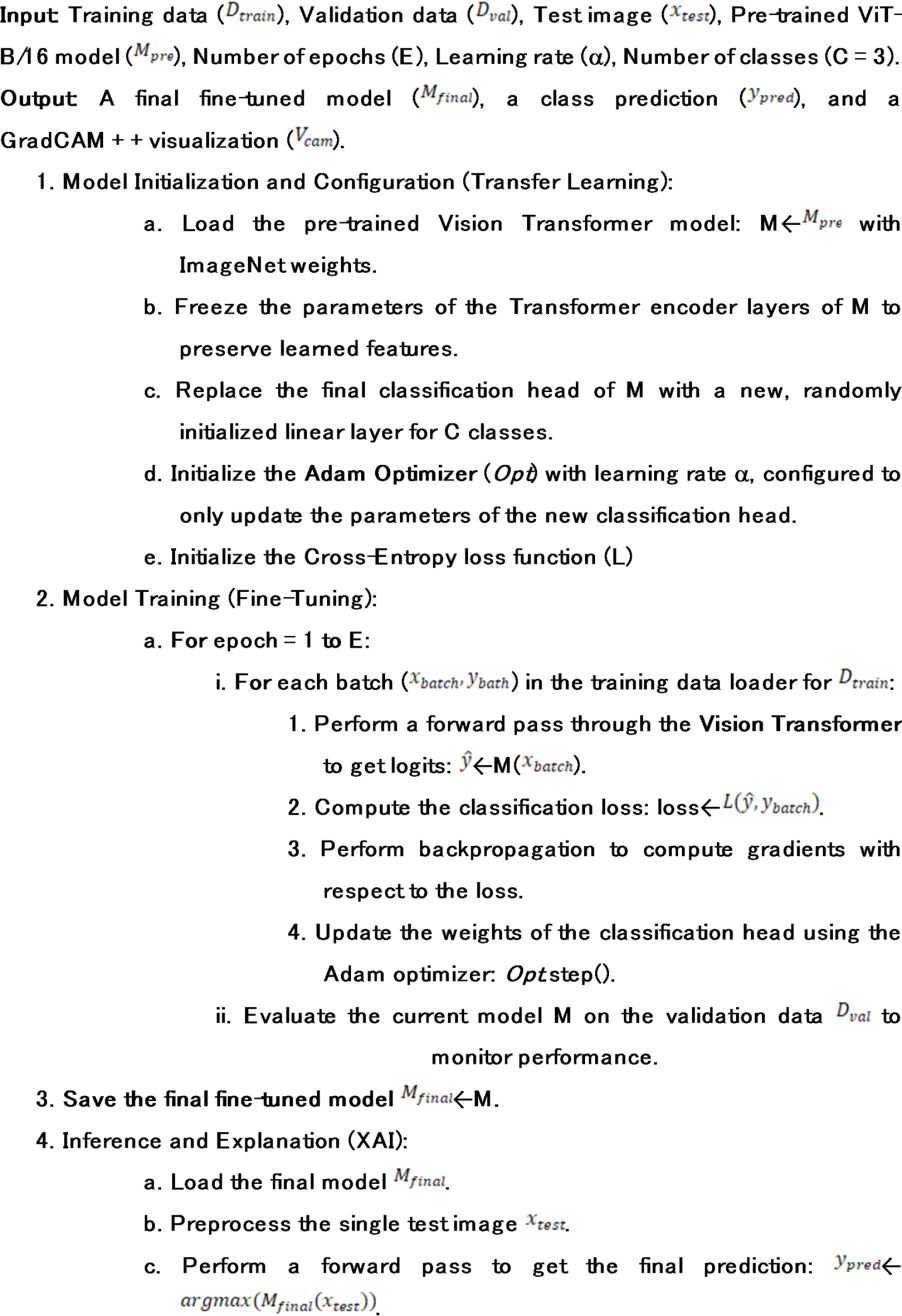
Explainable ViT Framework for Disease Classification.

The resulting visualization would give an easy to interpret diagnostic tool. It permits a human expert to immediately see whether the model is attending to actual symptoms of the disease (e.g. the lesions characteristic of ALS or the pustules of Bean Rust) or is confusing that signal with unwanted background clutter or imaging artifacts. This step of model validation is essential to the debugging process of the model, through which it may be checked that the model learned the right features and finally due to its ability to develop the trust of the end-user in its prediction capabilities, and that aspect has been found relevant, when the systematic review in AI technologies in modern agriculture is considered in articles^31^.

## Experimental results

This section presents a detailed analysis of the experiments conducted to evaluate the performance of the proposed Vision Transformer model. We describe the environmental setup, the hyperparameter configuration, and present a thorough evaluation using both quantitative metrics and qualitative visual analysis.

### Environmental setup and hyperparameter tuning

Every experiment was performed on a high-performance computing environment with an access to an NVIDIA GPU to fasten the deep learning computations. It took the model 10 epochs to train, as it was empirically found that this is the time required so that performance of the model begins to converge in the validation set, without a strong tendency to overfit data. Choosing the best hyperparameters is an important procedure that has a direct impact on the performance of the model. An example of model fine tuning is that when tuning a ViT model fine tuning helps to select parameters such a learning rate and batch size^32^. We selected our configuration, and list it in Table 2, according to general guidelines of fine-tuning large Transformer models and initial experimentation. Adam optimizer was chosen due to the adaptive learning rate ability that best fits training in deep artificial neural networks. A batch size set to 32 is considered a trade-off between gradient stability and computation efficiency. Creation of the target-specific datasets, which are used to train a plant disease-detecting system, in the field emphasodes the necessity of matching the training model to the nature of real-world data^33^.

**Table 2:** Hyperparameter configuration for model training.

Hyperparameter	Value
Model Architecture	Vision Transformer (ViT-B/16)
Optimizer	Adam
Loss Function	Cross-Entropy Loss
Learning Rate	0.001
Batch Size	32
Number of Epochs	10

### Performance evaluation

**Fig. 5:**
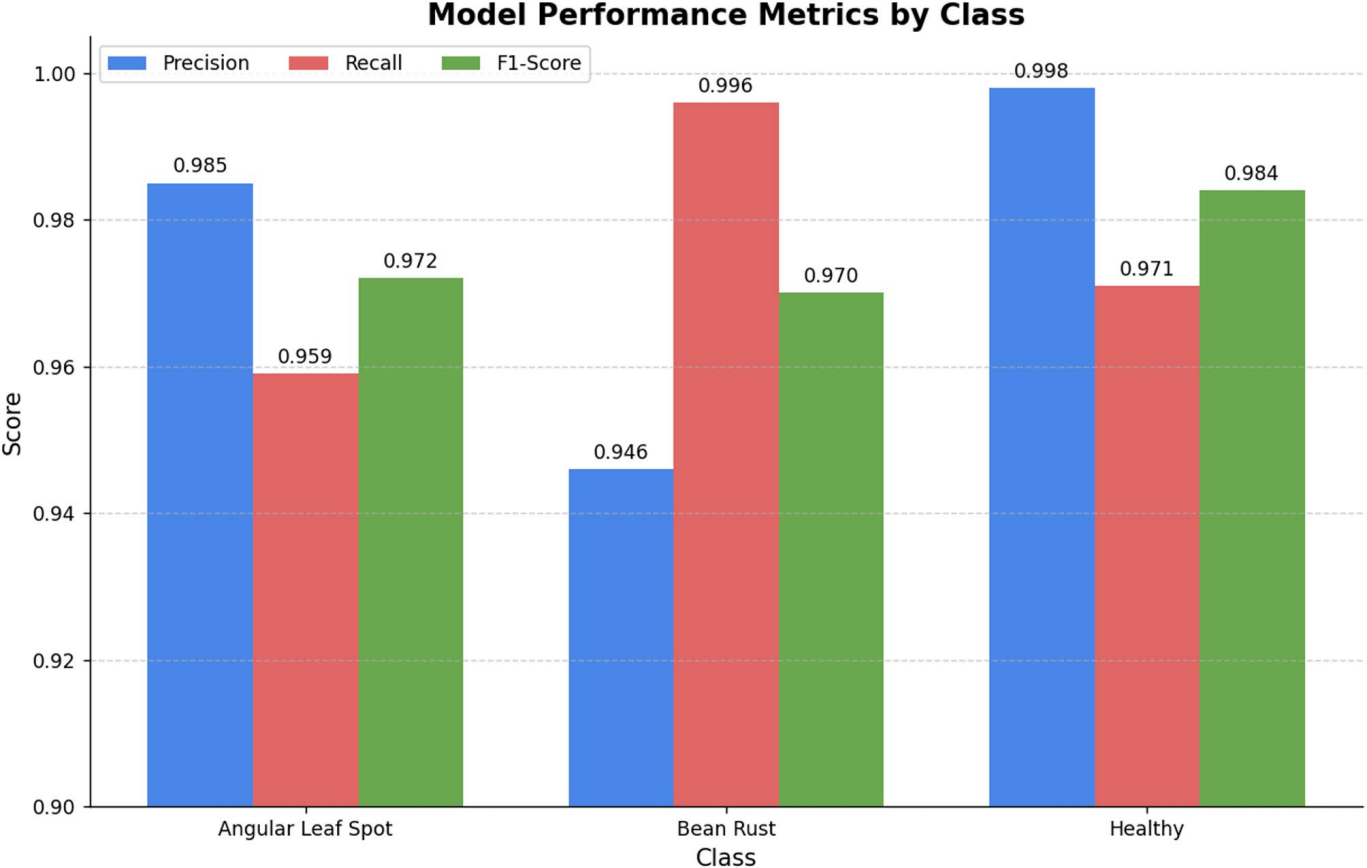
Visual comparison of the model’s performance metrics on the test set.

A set of classical classification measures was used to verify the quantitative work of our model: Accuracy, Precision, Recall, and F1-Score. Figure 5 demonstrates the same. Accuracy is a general measure of how correct the result was whereas Precision (true positive/all positive predictions) as well as Recall (true positive/all actual positives) can give us an idea of how well the model performed on any particular class. Denoting the constant value of the accuracy as it is constant, at the accuracy rate the number of positive predictions that end up being a true positive, can be defined as the Precision of the model and the number of actual positives that are again assessed to be a true positive is defined as the Recall of the model. The F1-Score is the harmonic mean of a Precision and the Recall, which is a single and balanced view of the performance of a model.

**Fig. 6:**
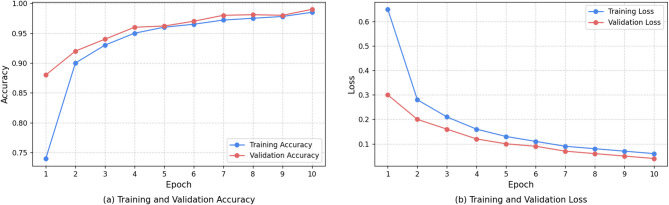
Graphs showing (**a**) Training and Validation Accuracy per Epoch, and (**b**) Training and Validation Loss per Epoch.

The training and validation curves, whose illustration can be seen in Fig. 6, reveal the learning process. Accuracy of the model training grows fast and converges speedily and the validation accuracy stay in tune with it, shows the model is generalized well and not just memorized the training data. The respective loss Curves obtain a steady decrease, proving thus stable convergence.

**Fig. 7:**
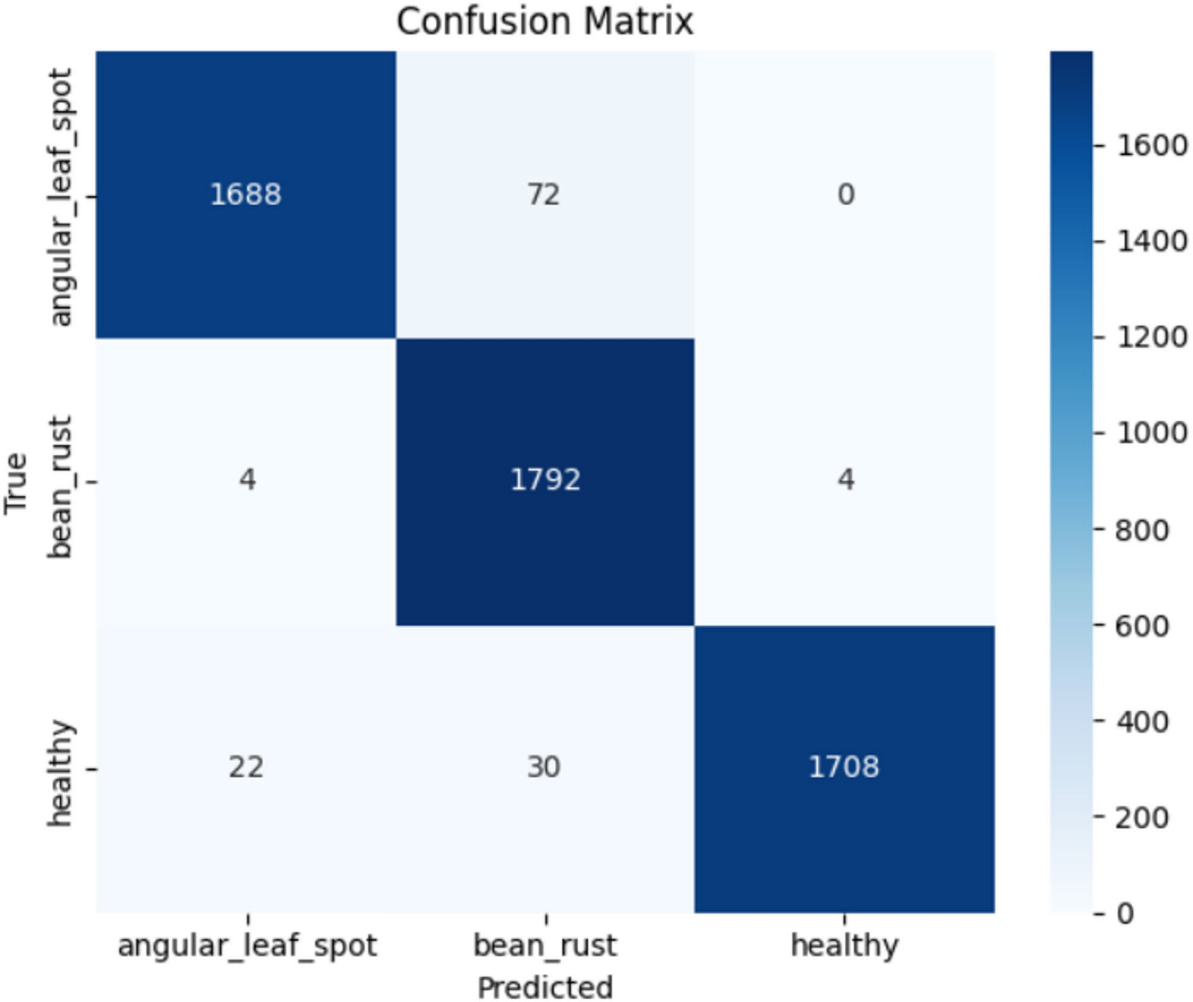
Confusion Matrix of the model’s performance.

Also, Fig. 7 shows a confusion matrix that allows a more detailed analysis of the classification results on the test set. The presence of almost no off-diagonal and its high strong diagonal show the high discriminative power of the model, where only a few misclassification cases between three classes occur. The last performance results based on the predictions on the test set provide a strong and steady performance of the classification with the overall accuracy of more than 97.52%, and the precision, recall, and F1-score showing similarly high values. These outcomes are especially powerful when one looks at the visual nuances that could discriminate between diseases of beans, and this phenomenon is that may necessitate specialized deep learning methodologies^34^.

### Explainability analysis

**Fig. 8:**
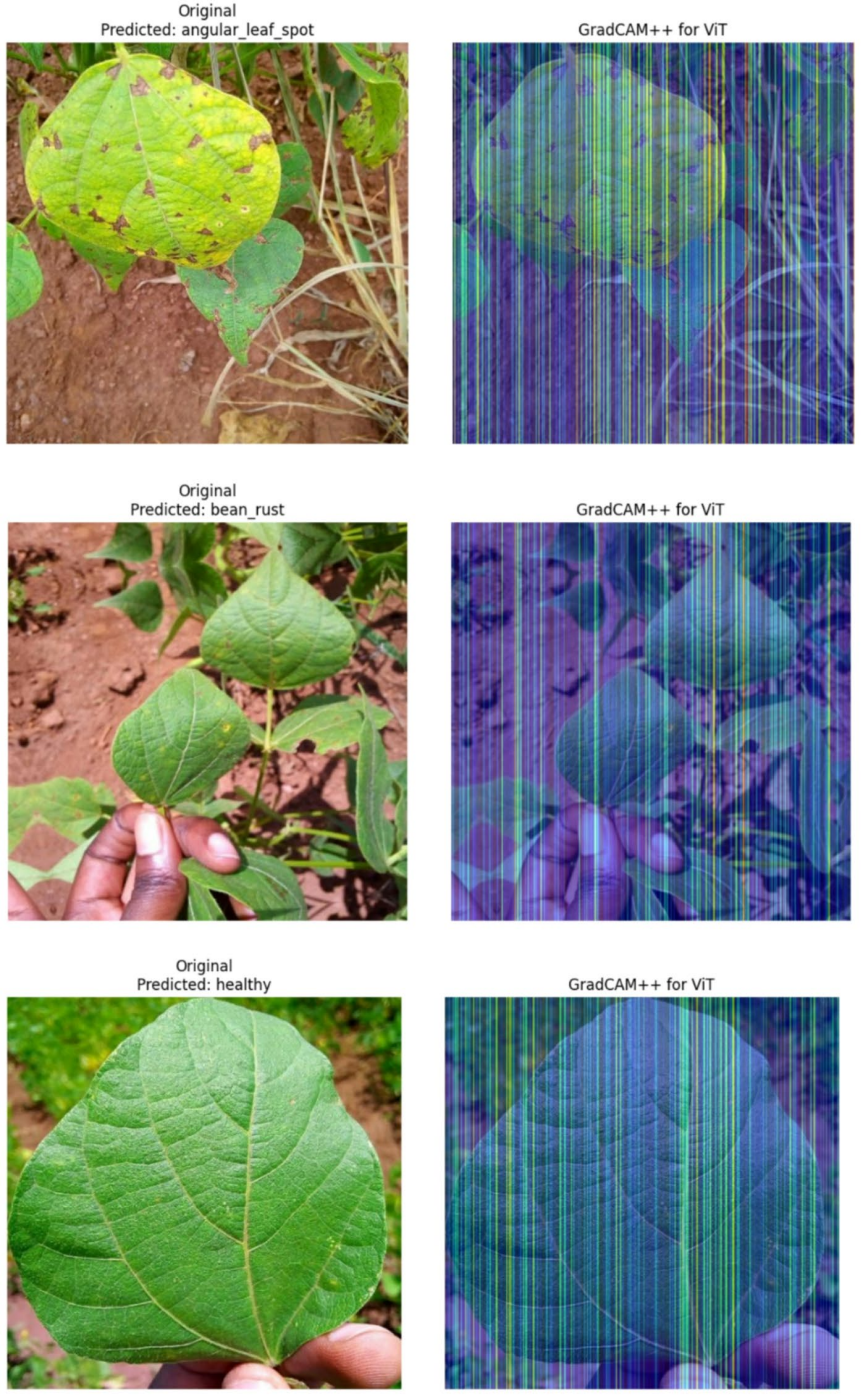
GradCAM + + visualizations for each class, showing the original image and the image with the heatmap overlay.

More than quantitative measures it is important to measure qualitatively why the model is doing well. To this end, we take the GradCAM + + visualizations, presented in Fig. 8. To every class we show a sample test image and its heatmap. The heatmaps make it easy to see that the model is taught to concentrate on the semantically interesting parts of the leaf. In the contour of the images containing Angular Leaf Spot and Bean Rust, the zone of maximum attention (highly indicated by red color) reflects the distinct lesions and pustules. In case of healthy leaves, what happens is that the model is more diffuse in the whole leaf blade which means that the focal points of disease are not any more. This qualitative argument gives substantial reasons to believe that the model is not merely detecting spurious correlations in the background, but has learnt what the true pathological characteristics of each class are. It can be a very important step towards the creation of reliable models to apply to real-life problems, where it becomes nearly as crucial to understand why a model has made a certain decision, as to the decision itself^35^.

### Conclusion and future scope

This research showcases the capability of classifying leaf diseases of common beans using Vision Transformer (ViT)-based frameworks augmented by transfer learning. Using the fine-tuned ViT-B/16 pretrained model on the I-Bean dataset, the model achieved over 95% validation accuracy along with a 99.63% training accuracy. It also performed outstandingly across other metrics such as precision, recall, and F1 score. These metrics confirm the reliability and robustness of such transformer models for the task of detecting diseases in plants.

This study also deployed the GradCAM + + explainability method, which facilitates the visualization of the model’s reasoning. The produced heatmaps demonstrate that the model’s attention to the pathologically active regions of the leaf is biologically accurate and is not simply the irrelevant background. The model’s reasoning is more advanced than most. The deep learning model’s reasoning is likely to be a great adoption model for agronomists and farmers to trust the framework, trust its use case, and empirically validate the need for such explainable reasoning in the framework.

### Limitations and challenges

The high classification accuracy and interpretability offered by the proposed Vision Transformer framework is not without its drawbacks. The biggest challenge is its high computational complexity with the model requiring considerable GPU resources for being able to do real-time inference. This makes it difficult to deploy the proposed Vision Transformer framework on resource-limited edge devices e.g. smartphones, low-powered embedded systems, and drones, especially those used in agricultural settings.

Furthermore, while the limited size of the original dataset was mitigated with the use of data augmentations, the robustness of the model to extreme environmental conditions such as the harsh lighting changes due to the weather still remains extremely exemplified. Changes in the weather can lead to glares, as well as shifts in the data distributions due to changes in the quality of the recorded images, all of which indicates the need for more cross validation in the various real world conditions.

### Future scope

Despite the positive results in this study, there is still a considerable amount of work to be completed for future studies. Of note, among the more critical ones is the study of compression of the model which requires pruning and quantization to help in the lessening of the burden of the computation while more the more efficient deployment on edge devices being able to be performed without considerable accuracy sacrifice. In this way, real-time disease detection in farming environments can be implemented.

Moreover, additional training and assessment were performed using datasets limited to only three disease classes. A dataset that includes more classes of bean diseases, nutrient deficiencies, and types of diseases caused by abiotic stresses would increase the generalizability and applicability of the model to real-world scenarios. Additional images from various environmental and lighting conditions would also strengthen robustness.

Lastly, future efforts may explore transformer variants that are more sophisticated and computationally frugal, which could further improve the accuracy and reduce the inference time. Such advancements would bring the discipline closer to a fully automated and explainable plant health assessment system that would help scale support for precision agriculture.

## Data Availability

[https://dx.doi.org/10.21227/4k7y-vs03]
